# Characterization of the spectrum of insecticidal activity of a double-stranded RNA with targeted activity against Western Corn Rootworm (*Diabrotica virgifera virgifera* LeConte)

**DOI:** 10.1007/s11248-013-9716-5

**Published:** 2013-06-08

**Authors:** Pamela M. Bachman, Renata Bolognesi, William J. Moar, Geoffrey M. Mueller, Mark S. Paradise, Parthasarathy Ramaseshadri, Jianguo Tan, Joshua P. Uffman, JoAnne Warren, B. Elizabeth Wiggins, Steven L. Levine

**Affiliations:** 1 Monsanto Company, 800 N Lindbergh Blvd., St. Louis, MO 63167 USA; 2 Monsanto Company, 700 Chesterfield Parkway W, Chesterfield, MO 63017 USA

**Keywords:** RNAi, Specificity, Coleoptera, Activity spectrum, Non-target organism

## Abstract

The sequence specificity of the endogenous RNA interference pathway allows targeted suppression of genes essential for insect survival and enables the development of durable and efficacious insecticidal products having a low likelihood to adversely impact non-target organisms. The spectrum of insecticidal activity of a 240 nucleotide (nt) dsRNA targeting the *Snf7* ortholog in Western Corn Rootworm (WCR; *Diabrotica virgifera virgifera*) was characterized by selecting and testing insects based upon their phylogenetic relatedness to WCR. Insect species, representing 10 families and 4 Orders, were evaluated in subchronic or chronic diet bioassays that measured potential lethal and sublethal effects. When a specific species could not be tested in diet bioassays, the ortholog to the WCR *Snf7* gene (DvSnf7) was cloned and corresponding dsRNAs were tested against WCR and Colorado potato beetle (*Leptinotarsa decemlineata*); model systems known to be sensitive to ingested dsRNA. Bioassay results demonstrate that the spectrum of activity for DvSnf7 is narrow and activity is only evident in a subset of beetles within the Galerucinae subfamily of Chrysomelidae (>90 % identity with WCR Snf7 240 nt). This approach allowed for evaluating the relationship between minimum shared nt sequence length and activity. A shared sequence length of ≥21 nt was required for efficacy against WCR (containing 221 potential 21-nt matches) and all active orthologs contained at least three 21 nt matches. These results also suggest that WCR resistance to DvSnf7 dsRNA due to single nucleotide polymorphisms in the target sequence of 240 nt is highly unlikely.

**Electronic supplementary material:**

The online version of this article (doi:10.1007/s11248-013-9716-5) contains supplementary material, which is available to authorized users.

## Introduction

RNA interference (RNAi) technology can achieve sequence-specific gene silencing in some insects by feeding double-stranded RNAs (dsRNAs) (Baum et al. 2007; Whyard et al. 2009; Swevers and Smagghe 2012). Sequence-specific gene silencing combined with the capability to suppress genes critical for insect survival suggests that dsRNAs can be used to develop insect control products that selectively target economically important pest species through ingestion of dsRNA and greatly reduces the likelihood of adversely affecting beneficial non-target organisms (NTOs). Whyard et al. (2009) demonstrated that even closely related species of the same genus can be selectively controlled with dsRNAs targeting regions of genes with no shared 19–21 nucleotide (nt) sequence between four *Drosophila* species. In addition to sequence specificity, additional barriers exist to oral toxicity in insects. These include potential degradation of the dsRNA prior to ingestion, barriers to cellular uptake, instability of the dsRNA within the insect following ingestion, and the inherent sensitivity of the organism to ingested dsRNA (Huvenne and Smagghe 2010; Allen and Walker 2012; Garbutt et al. 2013). It has been established with *Caenorhabditis elegans* that cellular uptake of a dsRNA and subsequent spreading involves the transmembrane receptor molecules SID-1 and SID-2 (Feinberg and Hunter 2003; Winston et al. 2007). Although SID-1 orthologs have been found in insect species, the mechanism of uptake of dsRNA by the midgut cells of insects and then spreading of targeted mRNA suppression to other tissues is still not well understood (Tomoyasu et al. 2008; Bolognesi et al. 2012; Miller et al. 2012). Recent studies on the tarnished plant bug (*Lygus lineolaris*) demonstrated that endonucleases present in saliva rapidly degrade dsRNA creating a barrier to an RNAi effect by oral exposure to dsRNA (Allen and Walker 2012). As summarized in a recent review by Huvenne and Smagghe (2010), insects display a wide range of sensitivities to ingested dsRNA, with the Coleoptera demonstrating significantly greater sensitivity than other insect Orders. Lepidoptera have demonstrated variable susceptibility to ingested dsRNA and high concentrations are required to elicit a response in this Order relative to Coleopterans (Huvenne and Smagghe 2010; Terenius et al. 2011). Additionally, rapid degradation of dsRNA in the hemolymph of *Manduca sexta* has been reported and attributed to nuclease activity, indicating that sensitivity to RNAi may be influenced by the instability of dsRNA within the insect (Garbutt et al. 2013).

Transgenic crops have been developed to express insecticidal dsRNAs and offer a new approach for agricultural pest control (Baum et al. 2007; Mao et al. 2007). For transgenic crops expressing an insecticidal dsRNA, lack of direct or indirect exposure provides an additional barrier for toxicity. Many phytophagous beetles (including members of the family Chrysomelidae) are known to be monophagous or oligophagous with plant hosts restricted to a genera, subfamily or family that are not related to the transgenic crop and are not found in agricultural ecosystems (Bernays 1988). Another mechanism potentially limiting environmental exposures is the length of the dsRNA. Bolognesi et al. (2012) recently demonstrated that a dsRNA must be of sufficient length (e.g. ≥60 bps) to result in efficacy against western corn rootworm (WCR, *Diabrotica virgifera virgifera*) and similar results were subsequently published by Miller et al. (2012) with *Tribolium*.

Previously, dsRNA targeting the *Snf7* ortholog in WCR, hereafter referred to as DvSnf7 dsRNA, was shown to be an efficacious target against WCR (Baum et al. 2007). Suppression of DvSnf7 mRNA and DvSnf7 protein levels in larval WCR occur in a time-dependent manner, with significant suppression of DvSnf7 mRNA after 24 h followed by suppression of the DvSnf7 protein, growth inhibition and mortality (Bolognesi et al. 2012). The *Snf7* protein is a class E vacuolar sorting protein functionally conserved in many organisms such as yeast (Vps32; Tu et al. 1993), humans (hSnf7 or CHMP4; Peck et al. 2004), mouse (mSnf7; Lee et al. 2007), fruit fly *Drosophila* (Shrub; Gao et al. 1999), nematode, *C. elegans* (CeVps32.2; Kim et al. 2011), and *Arabidopsis thaliana* (At2g19830 and At4g29160; Winter and Hauser 2006). *Snf7* belongs to the ESCRT (Endosomal Sorting Complex Required for Transport)–III complex, which has been shown to be essential for sorting of transmembrane proteins en route to lysosomal degradation through the endosomal-autophagic pathway in multiple organisms (Teis et al. 2008; Rusten et al. 2008; Lee and Gao 2008; Vaccari et al. 2009; Kim et al. 2011).

An assessment of spectrum of insecticidal activity for a pesticide is typically conducted during product development and is designed to characterize activity against a range of insect taxa that includes the target organism (Raybould 2006; Rose 2007; Romeis et al. 2013). Characterization of the spectrum of insecticidal activity, mode-of-action, as well as an understanding of environmental exposure levels and pathways provides important information that can narrow the scope of Tier 1 NTO testing for an ecological risk assessment (Romeis et al. 2008, 2013). Tier 1 NTO testing typically builds upon characterization of the activity spectrum and compliments specificity data by evaluating organisms that may be phylogenetically related and/or provide important functional roles (e.g., detritivores, predators, parasitoids, pollinators) in rigorous laboratory studies. The spectrum of activity for the DvSnf7 dsRNA was evaluated by selecting and assaying insects with varying degrees of phylogenetic relatedness to the DvSnf7 target in WCR. Insect species representing 10 families and 4 Orders were evaluated in continuous diet bioassays. Because lethality in WCR after ingestion of DvSnf7 dsRNA is typically not observed until 5–6 days after the initiation of exposure (Bolognesi et al. 2012), direct feeding studies ranged from 8 to 35 days and evaluated lethal and sub-lethal endpoints. When a phylogenetically-related insect species could not be tested in diet bioassays, the ortholog to the WCR *Snf7* gene was cloned and the correspondent dsRNA was tested against WCR and Colorado potato beetle (CPB, *Leptinotarsa decemlineata*) in 12-day diet bioassays. Due to their relatively high sensitivity to ingested dsRNA, WCR and CPB were used as model systems to evaluate the relationship between the minimum shared sequence length and efficacy (Baum et al. 2007).

## Materials and methods

### Species selection

Species were selected based upon phylogenetic relatedness to WCR, relevance of the tested organism as a surrogate for beneficial insects (e.g., predator, parasitoid), availability of published genome sequence data and the ability to test the organism in the laboratory in a sub-chronic or chronic dietary exposure. Since the WCR belongs to the Order Coleoptera, species from several families in this Order were assayed. Altogether, five coleopteran species representing four families were tested in direct feeding assays including: pink spotted lady beetle (*Coleomegilla maculata*); Mexican bean beetle (*Epilachna varivestis*); red flour beetle (*Tribolium castaneum*); and a carabid beetle species (*Poecilus chalcites*). WCR and southern corn rootworm (*Diabrotica undecimpunctata howardi*) LC_50_ values were previously determined in 12-day diet-incorporation bioassays and reported in Bolognesi et al. (2012). Four representatives of the Order Lepidoptera were assayed representing three families including; fall armyworm (*Spodoptera frugiperda*), corn earworm (*Helicoverpa zea*), European corn borer (*Ostrinia nubilalis*), and silkworm (*Bombyx mori*). An insect from the Order Diptera was not examined. Previously, a Dipteran species, (*Drosophila* spp.), was shown not to be sensitive to dietary dsRNA and insecticidal activity was only achieved by soaking larvae in solutions of dsRNA encapsulated within cationic liposomes (Whyard et al. 2009). Two species of Hymenoptera were assayed as surrogates for other Hymenoptera (e.g. honey bees) and represent two families; jewel wasp (*Nasonia vitripennis*) and eulophid wasp (*Pediobius foveolatus*). Honey bees (*Apis mellifera*) are routinely tested for ecological assessments and, therefore, were considered outside the scope of an activity spectrum assessment. Finally, one species from the Order Hemiptera, the insidious flower bug (*Orius insidiosus*) was assayed.

Species evaluated in the indirect feeding assays were selected based on their phylogenetic relatedness to WCR in accordance with the coleopteran phylogeny reported in Clark et al. (2001), Hunt et al. (2007) and Gillespie et al. (2008). WCR and CPB were tested with their conspecific (species specific) *Snf7* dsRNAs, DvSnf7 for WCR and LdSnf7 for CPB. Also, *Snf7* orthologs of five chrysomelid species: striped cucumber beetle (*Acalymma vittatum)*, bean leaf beetle (*Cerotoma trifurcata*), and black-margined loosestrife beetle (*Galerucella calamariensis*) from the subfamily Galerucinae, and Klamath weed beetle (*Chrysolina quadrigemina*) and yellow-margined leaf beetle (*Microtheca ochroloma*), from the subfamily Chrysomelinae, were evaluated. Additionally, the *Snf7* orthologous sequences from one other representative Galerucinae, the flea beetle *(Aphthona lacertosa)* was utilized for a multi-sequence alignment against DvSnf7.

### Insect sources

WCR and SCR eggs were obtained from Crop Characteristics, Inc. (Farmington, MN) and CPB eggs were obtained from French Agricultural Research (Lamberton, MN). *O. insidiosus* adults were purchased from the same source reported in Tan et al. (2011). *N. vitripennis* pupae were received in parasitized *Sarcophaga* (flesh fly) pupae from Ward’s Natural Science (Rochester, NY). *S. frugiperda* and *O. nubilalis* eggs were obtained from in-house cultures and *H. zea* eggs were obtained from Benzon Research Inc., (Carlisle, PA). *B. mori* eggs were received from Carolina Biological Supply Company (Burlington, NC). *C. maculata* eggs were obtained from a culture held at the USDA Agricultural Research Service (ARS) (Beltsville, MD). *P. foveolatus* pupae (in parasitized *E. varivestis*) and *E. varivestis* were obtained from the New Jersey Department of Agriculture (Philip Alampi Beneficial Insect Rearing Facility, Trenton, NJ). Adult *T. castaneum* (strain GA-1) were received from the USDA Agricultural Research Station (Manhattan, KS). *P. chalcites* eggs were obtained from a culture held at the USDA-ARS North Central Agricultural Laboratory (Brookings, SD). *A. vittatum* and *C. trifurcata* were collected locally and taxonomically verified in the laboratory. *G. calamariensis, A. lacertosa* and *C. quadrigemina* were purchased from Biological Control of Weeds (Bozeman, MT). *M. ochroloma* larvae (2nd instar) were purchased from a culture at Auburn University (H. Fadamiro Laboratory).

### Synthesis of DvSnf7 dsRNA

The DvSnf7 240 bp dsRNA was synthesized in vitro with the Ambion MEGAscript RNAi Kit (Life Technologies, Carlsbad, California). Two methods were used to synthesize the DvSnf7 dsRNA. These methods differed by the DNA template used for the in vitro transcription reaction. One method used a single PCR product of the DvSnf7 with opposing T7 polymerase promoters (TAATACGACTCACTATAGGG) at the 5′ ends of each strand. The DvSnf7 was amplified from a sequence-confirmed plasmid using a high fidelity polymerase and the single product was gel extracted using a Qiagen Gel Extraction Kit (Qiagen Inc., Valencia, CA, USA). This PCR product was used in the in vitro transcription reaction to create dsRNA which was purified according to manufacturer’s instructions. The second method used two clones with identical DvSnf7 sequences except that a single T7 promoter was at opposite ends of each of the DvSnf7 sequences. To create the clones, DNA fragments of the desired sequences with a single T7 polymerase promoter were cloned into the pUC19 vector (New England Biolabs, Ipswich, MA) between the EcoRI and HindIII restriction endonucleases recognition sites and were sequence confirmed. The clones were used in separate in vitro transcription reactions, after which the individual ssRNAs were combined, annealed to create dsRNA, and purified according to the manufacturer’s protocol. In vitro synthesized dsRNA was quantified by Nanodrop (Thermo Scientific, Wilmington, DE) at 260 nm and purity was evaluated by examining the 260/280 nm ratios. Products were examined by agarose gel electrophoresis to confirm integrity. DvSnf7 dsRNAs were confirmed to be biologically active and equipotent in diet bioassays with WCR or *D. undecimpunctata* *howardi* (data not reported).

### Sequencing of Snf7 orthologs and synthesis of respective dsRNAs

cDNA was prepared from RNA extracted from the following insects: *D. undecimpunctata howardi*, *A. vittatum, C. trifurcata*, *G. calamariensis, A. lacertosa,* *C. quadrigemina*, *M. ochroloma,* and CPB. For RNA extraction, adult insects were used with the exception of *D. undecimpunctata howardi*, *M. ochroloma,* and CPB, where 2nd instar larvae were used. RNA was extracted from insects using RNAeasy^®^ kit (Qiagen, Valencia, CA) and the cDNA was prepared using SuperScript™ First-Strand Synthesis kit (Life Technologies, Carlsbad, California). The 240 bp *Snf7* orthologous dsRNAs from *A. vittatum, C. trifurcata, G. calamariensis, C. quadrigemina, M. ochroloma* and CPB were synthesized using the methods outlined in the previous section. A fragment of the *Snf7* orthologs were amplified by PCR using cDNA from at least 3 individuals of each species as templates and degenerate primers designed based on the conserved flanking regions of the 240 bp DvSnf7 sequence. After sequence confirmation of the amplified products, specific primers flanking the 240 bp regions were designed. *Snf7* ortholog regions were amplified by PCR using these sequence specific primers and the PCR products were cloned into TOPO vector and sequence confirmed. The *Snf7*-240 bp regions were generated for each insect species from their respective plasmid clones.

### Bioassay methodology

For the majority of the insects tested, bioassays were conducted with diet-incorporation methodology and insects were fed ad libitum. Bioassays followed published methods or methods developed at the authors’ laboratory. Bioassays were initiated with the earliest life stages amenable to the assay design, and were of sufficient duration to result in sub-chronic or chronic exposures. For species used as surrogates for beneficial NTOs, consideration was given to selecting the life stage(s) with direct exposure to a plant expressed insecticidal dsRNA (e.g. pollen, root or leaf tissue). Diet concentrations were nominally 500–5,000 ng DvSnf7 dsRNA/mL or/g diet (see Table 1 for details), which greatly exceeds the mean 12-day WCR LC_50_ value of 4.3 ng/mL diet reported by Bolognesi et al. (2012). For comparison, tissue expression levels of DvSnf7 dsRNA in a greenhouse-grown transgenic corn event developed in-house were quantified with a maximum expression of 1.76 ng/g fresh weight in leaf tissue and 0.436 ng/g fresh weight root tissue and are two to three orders of magnitude below tested concentrations. The difference in diet concentrations of DvSnf7 dsRNA for the various species tested was due to the bioassays being conducted at different points in product development and is not related to the toxicity for an individual test species. Initially, concentrations were based upon a multiple for a 7-day WCR LC_50_ value which was consistently one order of magnitude greater than the 12-day LC_50_ value (data not reported), hence the order of magnitude difference spanning the test concentrations. Despite a range of concentrations tested, the levels of DvSnf7 dsRNA tested still provided a large (>250 times) margin between initial estimates of exposure and tested concentrations. Stability of dsRNA in insect diet matrices was evaluated and confirmed over the duration of the bioassays or between diet replacements (data not reported). These results are consistent with reported stability of siRNAs in insect diet (Borgio 2010; Upadhyay et al. 2011).

**Table 1:** Bioassay results from direct feeding studies with DvSnf7 dsRNA using insects from four Orders representing ten insect families

Order	Family	Subfamily	Species	Bioassay duration (days)	Endpoints	LC_50_^a^ or no observed effect concentration^b^ (ng/mL or g diet)
Coleoptera	Chrysomelidae	Galerucinae	*D. virgifera virgifera*	12	Survival	1.2^a^
	Chrysomelidae	Galerucinae	*D. undecimpunctata howardi*	12	Survival	4.4^a^
	Chrysomelidae	Chrysomelinae	*L. decemlineata*	12	Survival, Growth	5,000^b^
	Tenebrionidae	Tenebrioninae	*T. castaneum*	30	Survival, Growth	5,000^b^
	Coccinellidae	Coccinellinae	*C. maculata*	24	Survival, Growth, Development	3,000^b^
	Coccinellidae	Epilachninae	*E. varivestis*	28	Survival, Growth, Development	3,000^b^
	Carabidae	Harpalinae	*P. chalcites*	35	Survival, Growth, Development	5,000^b^
Hemiptera	Anthocoridae	Anthocorinae	*O. insidiosus*	9	Survival, Growth, Development	5,000^b^
Hymenoptera	Eulophidae	Entedoninae	*P. foveolatus*	21	Survival	3,000^b^
	Pteromalidae	Pteromalinae	*N. vitripennis*	20	Survival	5,000^b^
Lepidoptera	Noctuidae	Noctuinae	*S. frugiperda*	8	Survival, Growth	500^b^
		Heliothinae	*H. zea*	12	Survival, Growth	5,000^b^
	Crambidae	Pyraustinae	*O. nubilalis*	12	Survival, Growth	5,000^b^
	Bombycidae	Bombycinae	*B. mori*	14	Survival, Growth	5,000^b^

^a^LC_50_ data reported in Bolognesi et al. (2012)

^b^No observed effect concentration is equal to the maximum concentration tested in these bioassays. Concentrations ranged from 500 to 5000 ng/mL or ng/g diet and represent 250–2,500 times the maximum expected environmental concentration of DvSnf7 based on greenhouse expression levels

### Direct feeding bioassays

#### Hemiptera

##### O. insidiosus

Nymphs were exposed to DvSnf7 dsRNA incorporated into diet at 5,000 ng/g for a period of 9 days. Bioassay methods and conditions, including the production of nymphs in the laboratory, followed the methods described in Tan et al. (2011). An assay control and a positive control with potassium arsenate at 50 μg/g diet were included. Each treatment consisted of 40 individual nymphs hatched from the same batch on the same day. Encapsulated diets were replaced every 48 h and daily observations were made for survival and development to adulthood.

#### Hymenoptera

##### N. vitripennis

Assays were initiated with newly emerged adult females that were ≤24 h from first observed emergence. Wasps in the test group were fed 30 % (v/v) honey/water solution with DvSnf7 dsRNA at 5,000 ng/mL diet. Assay control groups were fed an untreated 30 % honey/water solution and the positive control group was fed 30 % honey/water solution with 100 μg potassium arsenate/mL diet. Wasps were incubated at 25 °C, 70 % RH (relative humidity) and 16L: 8D photoperiod. Diet treatments were administered in approximately 0.4 mL screened containers, renewed every 48 h when observations were made, and over a period of 20 days. Each dietary exposure treatment consisted of 25 wasps housed together in a vent-capped cell culture flask (Corning Inc., Corning, NY) and replicated three times.

##### P. foveolatus

Newly emerged adult wasps (≤30 h from the first observed emergence) were transferred to acclimation containers and allowed to feed on a 30 % (v/v) honey/water solution for 24 h prior to initiation of the test. Adults were exposed to DvSnf7 dsRNA incorporated into diet at 3,000 ng/mL for a period of 21 days along with an assay control and a positive control at 200 μg potassium arsenate/mL diet. Wasp pupae were incubated at a 25 °C, 70 % RH in a 14 L: 10 D photoperiod until adult emergence and for the duration of the assays. Test and control substances were administered to the wasps in approximately 0.4 mL screened containers and wasps were fed for the duration of the study. Each treatment consisted of 24–25 wasps housed together in a vent-capped cell culture flask (Corning Inc., Corning, NY) and replicated three times. The test and control diets were replaced every 48 h at which time mortality was recorded.

#### Lepidoptera

##### *S. frugiperda*, *H. zea* and *O. nubilalis*

Newly hatched larvae (≤30 h after the first observation of hatching) were exposed to DvSnf7 dsRNA in an agar-based diet at 500 ng/mL diet for *S. frugiperda* and 5,000 ng/mL for *H. zea* and *O. nubilalis*. The duration of the *S. frugiperda* bioassay was restricted to 8 days whereas the *H. zea* and *O. nubilalis* bioassays were 12 days. Treated diet was prepared by diluting the DvSnf7 dsRNA sample in 5 mL of purified water and then incorporating this solution with 20 mL of the agar-based multiple species diet (Southland, Lake Village, AR). The assay control contained an equal volume of purified water incorporated with 20 mL of the agar-based diet. Diets were dispensed into 128-well trays (Bio-Serv, Frenchtown, NJ) in 1.0 mL aliquots for *S. frugiperda* and *H. zea* and 0.5 mL aliquots for *O. nubilalis*. For *S. frugiperda*, 32 individual wells were prepared for the test and water control treatments. For *H. zea* and *O. nubilalis*, three replicates each with 16 insects were prepared for each treatment. Larvae were allowed to feed for the duration of the bioassay in an environmental chamber programmed at 27º C, at 60 % RH and a 14L: 10D photoperiod. Due to rapid growth, after 6 days *H. zea* were transferred to freshly treated diet to ensure sufficient diet for the 12-day assay. The number of surviving insects and their weights were recorded at the end of the bioassay. For *H. zea*, individual weights and mortality were also recorded at 10 days due to the onset of pupation.

##### B. mori

Newly-hatched larvae (≤24 h after the first observation of hatching) were fed fresh mulberry leaves for 3 days and then continuously exposed to DvSnf7 dsRNA in a 14-day mulberry (*Morus rubra*) leaf dip assay. Fresh mulberry leaves were submerged for 60 s in purified water containing DvSnf7 dsRNA at 5,000 ng/mL and 0.1 % Silwet L-77 surfactant to facilitate spreading of the solution on the leaf surface. Leaves submerged for 60 s with 0.1 % Silwet L-77 solution served as an assay control. After drying treated leaves were placed in a test arena (Solo 16 oz cups, Solo Cup Company, Lake Forest, IL) and infested with larvae. Each test and control treatment consisted of 5 replicates with 10 larvae per replicate. Freshly treated leaves were supplied every 2–3 days. A positive control using the tryptic-core of the Cry1Ab protein was applied to fresh mulberry leaves using a 5,000 ng/mL solution. Eggs and larvae were incubated at 27 °C, 70 % RH and a 14L: 10D photoperiod. Observations for mortality were made daily, dead larvae were removed, and larval weights were recorded at assay termination.

#### Coleoptera

##### C. maculata

First instar larvae (≤48 h after the first observation of hatching) were continuously exposed to an agar-based pollen diet with DvSnf7 dsRNA at a 3,000 ng/g for a period of 24 days. An assay control and a positive control at 100 μg potassium arsenate/g of diet were included. Newly hatched larvae were acclimated to test conditions for 24 h and fed corn earworm (*H.zea)* eggs to reduce cannibalism prior to initiation of the test. Larvae were maintained individually in Petri dish (60 mm × 15 mm, BD Falcon, Franklin Lakes, NJ) test arenas and approximately 0.15 mL of test or control diet was administered every 48–72 h. Each treatment consisted of four replicates of 20 larvae for a target of 80 insects per treatment. Bioassays were incubated at a 27 °C, 70 % RH and a 14L: 10D photoperiod. Observations for mortality and larval development were made at each diet replacement until pupation. Once larvae had pupated, observations were made daily for adult emergence and adults were weighed within 32 h of eclosion.

##### E. varivestis

First instar larvae (≤24 h after the first observation of hatching) were fed an agar-based diet containing DvSnf7 dsRNA at 3,000 ng/mL for a period of 28 days. An assay control treatment and two positive control treatments with at 14 and 28 μg potassium arsenate/mL diet were included. Larvae were housed individually in 128-well bioassay trays (Bio-Serv Frenchtown, NJ), and wells contained 0.25 mL of the treatment diets. Each treatment consisted of three replicates with 16 larvae per replicate and were incubated at 27° C, 70 % RH and a 14L: 10D photoperiod. Observations for mortality and development were made every 7 days during each diet replacement. Survival, development stage and weight were recorded on day 28.

##### T. castaneum

Newly hatched larvae (≤30 after the first observation of hatching) were exposed to DvSnf7 dsRNA incorporated into a wheat flour diet (water content 20 %) at 5,000 ng/g over 30 days and followed methodology described in Whyard et al. (2009). An assay control and positive control treatments with 20 and 100 μg potassium arsenate/g diet were included. *T. castaneum* were cultured and tested at 30° C, 75 % RH and in total darkness. To collect neonates of a known age, eggs were sieved (# 50 sieve) from the laboratory culture and allowed to hatch in a separate container. Approximately 0.1 g of flour diet was administered to larvae in a 48 well plate. For the test and control treatments, three replicates with a target number of 50 larvae were tested per replicate. The positive control treatment was conducted with two replicates, with a target number of 55–65 larvae per replicate. Larvae were placed individually into each well. Because *T. castaneum* larvae burrow into the flour diet, test and control diets were not replaced. Observations for survival, developmental stage and weights were made at the end of the test.

##### P. chalcites

First instar larvae (≤24 h after the first observation of hatching) were fed an agar-based diet with a DvSnf7 dsRNA at 5,000 ng/g diet over 35 days following Duan et al. (2005). An assay control and a positive control at 200 μg potassium arsenate/g diet were included. Larvae were placed individually into test arenas and incubated at 27 °C, 70 % RH and a of 14L: 10D photoperiod. Each test arena was a 31 mL (1 oz) plastic portion cup, with a snap-on lid, filled with approximately 15 mL (1 tablespoon) of top soil that served as protective microhabitat for the larvae and developing pupae. Approximately 0.15 mL of test and control diet was administered every 24–48 h until pupation. Test and control treatments consisted of three replicates with a target of 25–30 larvae per replicate. Observations for mortality and development were made at each diet replacement until pupation, after which daily observations were made until adult emergence and adults were weighed within 32 h of eclosion.

### Reciprocal feeding bioassays with WCR and CPB

The LC_50_ value for LdSnf7 dsRNA against CPB was estimated with concentrations ranging from 1.6 to 200 ng dsRNA/mL diet, with a twofold separation factor, and an assay control. Previously the 12-day WCR LC_50_ with DvSnf7 dsRNA was reported (Bolognesi et al. 2012). DvSnf7 dsRNA was tested against CPB at a single concentration of 5,000 ng/mL diet and LdSnf7 was tested against WCR at a single concentration of 15,000 ng/mL diet with a target number of 64 larvae per treatment. Treatments were prepared by diluting the dsRNA with purified water and incorporating the dilution into an agar-based *D. undecimpunctata* *howardi* or CPB diet (Bio-Serv, Frenchtown, NJ) on the day of preparation. WCR diet was prepared utilizing *D. undecimpunctata howardi* diet modified as described by Bolognesi et al. (2012). Diet for WCR was dispensed in 0.25 mL aliquots into 48 well plates (BD Falcon, Franklin Lakes, NJ) and diet for CPB was dispensed in 0.5 mL aliquots into a 128-well tray (Bio-Serv, Frenchtown, NJ). As a positive control, DvSnf7 dsRNA was tested against WCR and LdSnf7 was tested against CPB at a single effect concentration of 50 ng/mL diet with a target number of 32 and 24 larvae, respectively. WCR bioassays were incubated in a dark environmental chamber programmed at 25º C, 70 % RH and CPB bioassays were incubated at 27º C, 60 % RH with a 14L: 10D photoperiod. At bioassay termination, the number of insects infested and the number of surviving insects in each treatment were recorded.

### Indirect feeding bioassays

Several Chrysomelid species closely related to WCR, such as members of the Galerucinae (*A. vittatum*, C*. trifurcata, and G. calamariensis*) and Chrysomelinae (*C. quadrigemina* and *M*. *ochroloma*) subfamilies, are difficult to maintain and bioassay in the laboratory. To evaluate the potential toxicity of DvSnf7 dsRNA against these species, WCR and CPB were used as model systems and fed heterospecific (different species) dsRNA targeting the *Snf7* ortholog mRNA in *A. vittatum*, *C. trifurcata*, *G. calamariensis, C. quadrigemina* and *M*. *ochroloma* in diet bioassays. WCR and CPB were tested in 12-day diet-incorporation bioassays with a single dietary concentration of heterospecific dsRNA as summarized in Tables 3 and 4. The dietary concentrations of heterospecific dsRNAs were prepared at approximately 50–1,000 times the conspecific dsRNA 12-day LC_50_ values for WCR and CPB. WCR and CPB bioassays used a target number of 118 and 64 larvae, respectively, for the test treatment and 100 larvae for the control treatment. Insect handling procedures, diet preparation and environmental conditions were as previously described for WCR and CPB. At bioassay termination, the number of insects infested and the number of surviving insects in each treatment were recorded.

### Sequence alignments and phylogenetic tree

Orthologous *Snf7* sequence alignments and a phylogenetic tree were prepared using Jalview Version 2.8 (Clamp et al. 2004; Waterhouse et al. 2009) to demonstrate the divergence of the *Snf7* sequence across selected Coleoptera. Alignments were analyzed using the ClustalWS algorithm and colored based upon the percentage of sequence identity. The phylogenetic tree and average distances between species were calculated based upon the percent identity of the aligned sequences.

### Data analysis

Continuous data (e.g., growth) was analyzed for normality with Sharpiro-Wilks Test (α = 0.05) and for homogeneity of variance with Levene’s test (α = 0.05). All continuous data was shown to be normally distributed and have homogeneous variance. Therefore, all continuous endpoints were compared against the appropriate control with a parametric *t* test (α = 0.05). Binary endpoints (survival and development) were evaluated with Fisher’s Exact test (α = 0.05). LC_50_ values were estimated using PROC PROBIT within the SAS software and the OPTC option was used to correct for the natural rate of mortality (SAS Institute Inc., 2002–2008). Endpoints are reported as means with standard errors.

## Results and discussion

RNAi insect control technology has the potential for high taxonomic specificity and to target only one or a group of closely related species (Whyard et al. 2009). A benefit of high taxonomic specificity is that only species closely related to the targeted pest species will have the potential to be affected by the dsRNA (Romeis et al. 2013). Consequently, characterization of the spectrum of activity for an insecticidal dsRNA should evaluate species over a range of phylogenetic relatedness to the target species. This approach will enable a relevant and reliable characterization of the spectrum of activity that can support a NTO risk assessment including threatened and endangered species assessments. Additionally, characterizing the spectrum of insecticidal activity helps to determine the scope of NTO testing and the selection of appropriate test species for Tier 1 testing that addresses specific protection goals (Romeis et al. 2008). As recently discussed by Romeis et al. (2013), confidence in results from NTO studies will increase if test organisms are phylogenetically related and/or are tested in combination with species that provide valued ecosystem services.

The spectrum of activity for the DvSnf7 dsRNA was evaluated by selecting and assaying insects of varying phylogenetic relatedness to the target WCR. Insect species representing 10 families and 4 Orders were evaluated in continuous feeding diet bioassays with the DvSnf7 dsRNA. The four Orders included species from Hemiptera, Hymenoptera, Lepidoptera and Coleoptera. The largest number of species tested was from the Coleoptera since the most phylogenetic resolution, required to determine the spectrum of activity, can be achieved by testing within the Order of the target species. Dietary delivery provides the most ecologically relevant route of exposure for a plant incorporated insect control product, regardless of the susceptibility of a given insect to ingested dsRNA, and diet bioassays have been recognized as a valid approach to evaluating the specificity and potential adverse effects to NTOs for insecticidal dsRNAs (Burand and Hunter 2013). Bioassays ranged from 8 to 35 days and evaluated lethal and sublethal endpoints to adequately characterize the potential for adverse effects. Based upon the biology of the individual test species, the endpoints measured, and the established mechanism of action and timing of adverse effects of DvSnf7 dsRNA in WCR as reported in Bolognesi et al. (2012), the duration of these bioassays can be considered sub-chronic or chronic exposures and sufficient to detect potential adverse effects in non-target species.

### Hemiptera

The potential for DvSnf7 dsRNA to adversely effect survival and development of *O. insidiosus* was evaluated in a 9-day diet incorporation assay at a nominal concentration of 5,000 ng/g diet. Treatment and the assay control nymphs exhibited 100 % survival and adult emergence indicating no impact of DvSnf7 dsRNA with average development time to adults of 10.7 ± 0.1 and 10.6 ± 0.1 days, respectively, which are comparable with development times reported by Tan et al. (2011) (Table 1; Online Resource 1, Figure A). In the positive control treatment, mortality was observed at day 1 and reached 88 % by day 9. Only 13 % of these nymphs emerged as adults and average development time was 18.8 days confirming the effectiveness of the test system to detect toxic effects. Observing no adverse effect is consistent with another hemipteran species, *L. lineolaris*, where it was demonstrated that salivary endonucleases create a barrier to an RNAi effect with oral dsRNA delivery (Allen and Walker 2012).

### Hymenoptera

Adult parasitic wasps are known to consume plant-derived food sources such as pollen and nectar (Jervis et al. 1993). Therefore, the potential for DvSnf7 dsRNA to effect survival of adult *N. vitripennis* and *P. foveolatus* were evaluated in 20- and 21-day bioassays, respectively. No adverse effect from DvSnf7 dsRNA was evident on *N. vitripennis* at 5,000 ng/mL. Mean survival in the test and assay control groups was not significantly different (*p* > 0.05) and was 95 ± 4 and 97 ± 1 %, respectively, with 59 ± 4 % mean survival in the positive control, with a significant effect on mortality (*p* < 0.05) (Table 1; Online Resource 1, Figure B). No mortality was observed with *P. foveolatus* at 3,000 ng/mL diet or the control indicating no impact of the DvSnf7 dsRNA (Table 1); however, in the positive control treatment mortality reached 100 % by day 16. A comparison of the *N. vitripennis* orthologous 240 nt *Snf7* (UniGene sequence Nvi#S49011431) to the DvSnf7 dsRNA sequence showed no contiguous sequence match greater than 14 nt and an overall shared sequence identity of 71 % (Online Resource 2). The lack of a 21 nt match is consistent with the finding of no activity. However, it is unknown if *N. vitripennis* is susceptible to ingested dsRNA.

### Lepidoptera

The potential for DvSnf7 dsRNA to effect growth and survival of three Lepidoptera families was evaluated in diet incorporation or leaf dip bioassays. In an 8-day diet incorporation assay with *S. frugiperda*, 100 % survival was observed in both the control and test treatment exposed at 500 ng/mL diet DvSnf7 dsRNA. Mean *S. frugiperda* larval body weights for the test and assay control treatments were not significantly different (*p* > 0.05) and were 137 ± 8 and 145 ± 11 mg, respectively, indicating no adverse effects of the DvSnf7 dsRNA (Table 1; Online Resource 1, Figure C). In the 12-day assay with *H. zea*, 100 % survival was observed in the test and assay control treatments exposed at 5,000 ng/mL diet DvSnf7 dsRNA. Mean individual *H. zea* larval body weights at 10 days in the test and control treatments were not significantly different (*p* > 0.05) and were 310 ± 3 and 298 ± 2 mg, respectively, indicating no impact of DvSnf7 dsRNA (Table 1;Online Resource 1, Figure D). At 12 days, there was 85 % larval survival in the DvSnf7 dsRNA treatment and 80 % larval survival in the water control. 12-day *H. zea* larval weights were not significantly different (*p* > 0.05) with mean values of 277 ± 8 in and 282 ± 9 mg in the test and control treatments, respectively. Similar to the *H. zea* results, there was no significant effect (*p* > 0.05) on *O. nubilalis* survival with 100 and 94 % survival in the control and test treatment at 5,000 ng/mL diet DvSnf7 dsRNA, respectively (Table 1; Online Resource 1, Figure E). *O. nubilalis* larval body weights were not significantly different (*p* > 0.05) with mean weights of 47 ± 0.8 and 49 ± 0.2 mg for the test and control treatments, respectively. (Online Resource 1, Figure F).

Mean survival of *B. mori* in a 14-day leaf-dip bioassay with DvSnf7 dsRNA at 5,000 ng/mL was 98 ± 3 % compared to 92 ± 10 % control survival for larvae fed the 0.1 % Silwet L-77-only dipped leaves indicating no impact of the dsRNA on the measured endpoints (Table 1; Online Resource 1, Figure G). Mean *B. mori* body weights at test termination were 362 ± 18 and 313 ± 20 mg for the test and assay control treatments, respectively (Online Resource 1, Figure H). The effectiveness of the leaf dip bioassay to detect toxic effects under these exposure conditions was verified with a Cry1Ab positive control. Survival and growth in the Cry1Ab positive control treatment were 22 ± 5 % and mean larval weight of 194 ± 51 mg, respectively, and were significantly lower than the control (*p* < 0.05). A comparison of the *B. mori* orthologous 240 nt *Snf7* (UniGene sequence Bmo#S58881086) sequence to DvSnf7 dsRNA showed no contiguous sequence match greater than 15 nt and an overall shared sequence identity of 66 % (Online Resource 3). RNAi studies with Lepidoptera have demonstrated varying degrees of success. In general, very high concentrations of dsRNA have been required to elicit a response in feeding studies with Lepidoptera species (Terenius et al. 2011). The Lepidoptera species in this study were exposed to DvSnf7 dsRNA at concentrations that were significantly higher than measured DvSnf7 dsRNA expression in a corn plant.

### Coleoptera

In total, seven species of Coleoptera representing four families were tested in direct feeding assays (Table 1). The biological activity of DvSnf7 dsRNA against the target WCR and *D. undecimpunctata* *howardi* was previously characterized in 12-day diet incorporation bioassays (Bolognesi et al. 2012) (Table 1; Online Resource 1, Figure I). Comparable efficacy was observed with WCR and *D. undecimpunctata howardi* and this result was predicted considering the high degree of sequence identity (>98 %; Table 2) between these two closely related species (Bolognesi et al. 2012).

**Table 2:** Percent identity, numbers of single nucleotide polymorphisms (SNPs) and number of 21 nt matches of *Snf7* orthologs from ten Coleoptera in the families Chrysomelidae and Tenebrionidae

Species	Subfamily, Tribe	Percent identity to DvSnf7 dsRNA	No. SNPs	No. 21 nt matches (or longest contiguous sequence)
*D. virgifera virgifera*	Galerucinae, Luperini	100	0	221
*D. undecimpunctata howardi*	Galerucinae, Luperini	98.8	3	186
*A. vittatum*	Galerucinae, Luperini	95.0	12	69
*C. trifurcata*	Galerucinae, Luperini	90.8	22	18
*G. calamriensis*	Galerucinae, Galerucini	90.8	22	3
*A. lacertosa*	Galerucinae, Alticini	81.7	44	0, (17 nt)
*C. quadrigemina*	Chrysomelinae, Chrysomelini	82.1	43	0, (19 nt)
*M. ochroloma*	Chrysomelinae, Chrysomelini	79.6	49	0, (19 nt)
*L. decemlineata*	Chrysomelinae, Chrysomelini	78.3	52	0, (14 nt)
*T. castaneum*	Tenebrioninae, Tribolini	72.1	67	0, (11nt)

The inclusion of a representative of the Tenebrionidae is to provide context for the degree of sequence divergence outside the Chrysomelidae

The potential for DvSnf7 dsRNA to effect survival of *C. maculata* was evaluated in a 21-day diet bioassay at 3,000 ng/g diet. No mortality was observed in the dsRNA treatment or the assay control indicating no impact of DvSnf7 dsRNA (Table 1). The mean time to adult emergence were 19.6 ± 1.2 and 19.6 ± 1.5 days for the test and control DvSnf7 dsRNA, respectively, indicating no adverse effects on development from ingestion of DvSnf7 dsRNA (Table 1; Online Resource 1, Figure J). Mean adult weights for the DvSnf7 dsRNA and the assay control treatments were not significantly different (*p* > 0.05) with mean values of 10.1 ± 0.1 and 10.5 ± 0.2 mg, respectively (Online Resource 1, Figure K). In the positive control treatment no adults emerged by test termination and only 11 % of the larvae developed to the pupal stage.

The potential for DvSnf7 dsRNA to effect survival of *E. varivestis* was evaluated in a 28-day diet bioassay at 3,000 ng/mL diet. No differences in mean survival were observed between the DvSnf7 dsRNA treatment and the water only controls, with the test and control treatments exhibiting 92 ± 7 and 92 ± 4 % survival, respectively (Table 1; Online Resource 1, Figure L). In the DvSnf7 dsRNA treatment, 100 % of the surviving larvae reached the 4th instar larval stage and 98 % of *E. varivestis* larvae reached the 4th instar stage in the control. Mean weight for the DvSnf7 dsRNA and assay control larvae were not significantly different (*p* > 0.05) and were 20 ± 1 and 19 ± 1 mg, respectively (Online Resource 1, Figure M). The positive control treatments demonstrated significant effects on survival from both the 14 and 28 μg potassium arsenate/mL diet treatments with 42 ± 14 and 8 ± 4 % survival, respectively.

*T. castaneum* have been commonly used in studies utilizing RNAi methodology employing dsRNA by injections (Tomoyasu et al. 2008; Miller et al. 2012). However, RNAi activity has also been observed by ingestion of dsRNA in a 7-day bioassay (Whyard et al. 2009). The potential for activity of DvSnf7 dsRNA on survival of *T. castaneum* was evaluated in a 30-day diet bioassay at 5,000 ng/g diet. No mortality was observed in the DvSnf7 dsRNA treatment and the assay control. In the positive control treatment, no adults emerged by test termination and only 22 % of the larvae developed to the pupal stage (Table 1). A comparison to the orthologous 240 nt *Snf7* in *T. castaneum* (UniGene sequence Tca#S32316245) to the DvSnf7 dsRNA sequence showed no contiguous sequence match greater than 11 nt and an overall 72 % shared sequence identity (Table 2; Online Resource 4).

The potential for DvSnf7 dsRNA to effect survival of *P. chalcites* was evaluated in a 35-day diet bioassay at 5,000 ng/g diet. Survival in the DvSnf7 dsRNA treatment and water control were not significantly different (*p* > 0.05) with 89 ± 8 and 90 ± 5 % survival, respectively(Table 1; Online Resource 1, Figure N). Additionally, mean development time to adult emergence was not significantly different (*p* > 0.05) with 29 ± 1 and 30 ± 1 day for the DvSnf7 dsRNA and assay control treatment, respectively (Online Resource 1, Figure O). Mean adult weights were not significantly different (*p* > 0.05) and were 41 ± 1 mg for the DvSnf7 dsRNA treatment and 40 ± 1 for the assay control (Online Resource 1, Figure P). The positive control treatment demonstrated significant effects on development and survival with no adults emerging in this treatment and the mean survival at test termination was 64 ± 3 %.

In a series of well designed experiments Whyard et al. (2009) demonstrated that several different insect taxa were selectively killed when fed conspecific dsRNAs targeting vATPase transcripts. For example, when *T. castaneum* were fed a heterospecific dsRNA targeting the pea aphid (*Acyrthosiphon pisum*), and *A. pisum* were fed the heterospecific dsRNA targeting *T. castaneum*, no activity of the dsRNA was observed. Conversely, both *T. castaneum* and *A. pisum* were sensitive when fed their conspecific dsRNAs. Taking this approach one step further, it was demonstrated that specific activity could be achieved at the species level when variable regions of genes were targeted showing the tremendous potential for specificity that could be achieved with dsRNA (Whyard et al. 2009). Using a similar approach, Baum et al. (2007) examined the potential for species specificity with WCR and CPB using dsRNAs that targeted V-ATPase subunits A and E for each species. The V-ATPase subunit A target sequences from CPB and WCR share 83 % nucleotide-sequence identity whereas the V-ATPase subunit E target sequences from these organisms share 79 % nucleotide-sequence identity (Baum et al. 2007). Ingestion of the heterospecific dsRNA that targeted V-ATPase subunits A and E caused mortality in both WCR and CPB but with approximately tenfold less activity compared to the conspecific dsRNA. Activity of the heterospecific dsRNA was expected because of the presence of multiple 21 nt shared sequence over the targeted portion of the gene for these two species.

**Fig. 1:**
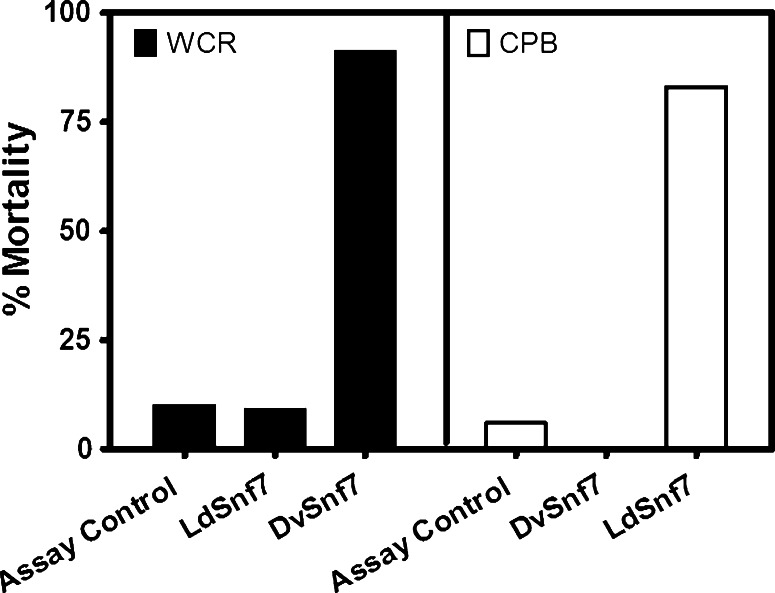
WCR and CPB bioassays demonstrate specificity between two closely related species in the family Chrysomelidae. Conspecific and heterospecific *Snf7* dsRNAs from WCR and CPB were tested at in 12-day diet incorporation bioassays. LdSnf7 and DvSnf7 dsRNAs were tested against WCR at concentrations of 15,000 and 50 ng/mL diet, respectively. DvSnf7 and LdSnf7 dsRNAs were tested against CPB at concentrations of 5,000 and 50 ng/mL diet, respectively

In the present study, we repeated the experimental approach used by Baum et al. (2007) with WCR and CPB. CPB demonstrated a concentration-dependent effect after ingestion of its conspecific dsRNA targeting the *Snf7* ortholog in CPB (i.e., LdSnf7). The 12-day LC_50_ value was estimated to be 19 ng/mL diet (95 % interval of 11–29 ng/mL diet; slope 1.6), which is comparable to and only about 4-times higher than the mean 12-day LC_50_ value for DvSnf7 dsRNA against WCR reported by Bolognesi et al. (2012). At an LdSnf7 dsRNA diet exposure level of 15,000 ng/mL there was not a significant difference in WCR mortality compared to the assay control (*p* > 0.05; Fig. 1). Similarly, at a DvSnf7 dsRNA diet exposure level of 5,000 ng/mL there was no significant difference in CPB mortality compared to the assay control (*p* > 0.05; Fig. 1). Additionally, there was no significant effect on CPB growth with mean control and test weights of 8.5 ± 0.2 and 8.2 ± 0.4 mg, respectively (*p* > 0.05). However, a high level of mortality was observed when the conspecific dsRNAs at a concentration of 50 ng/mL diet were tested against each species (Fig. 1). These reciprocal feeding assays, conducted with two closely related species, exemplify the high level of taxonomic specificity that can be achieved with this 240 nt DvSnf7 dsRNA.

### Bioinformatics and indirect feeding bioassays

**Fig. 2:**
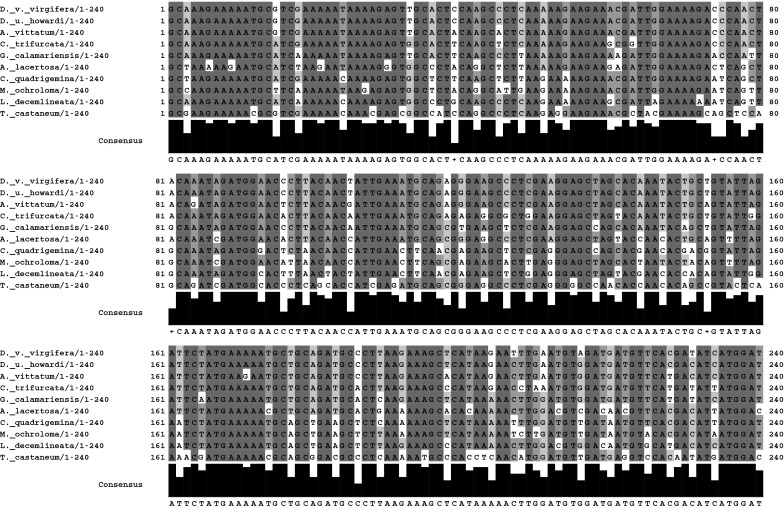
ClustalWS multispecies alignment of *Snf7* 240 nt orthologs in selected Chrysomelid species. The coloring is based upon the percent of shared sequence identity. A representative of the family Tenebrionidae (*T. castaneum*) was used to anchor the tree. Created using Jalview Version 2.8. The alignment order parallels that shown for the phylogenetic tree in Fig. 3

**Fig. 3:**
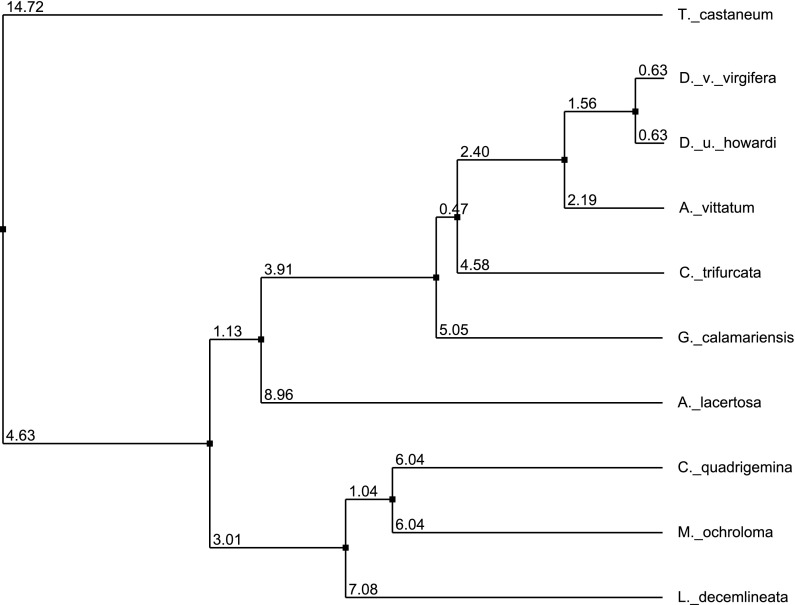
Average distance phylogenetic tree of *Snf7* orthologs based upon percent identity and ClustalWS alignment. The tree illustrates the rapid divergence of *Snf7* sequences within the coleopteran family Chrysomelidae. A representative of the family Tenebrionidae (*T. castaneum*) was used to anchor the tree. The tree was calculated on distance matrices using the percent identity between sequences (represented numerically below) and created using Jalview Version 2.8

In using RNAi as an insect control technique, the chosen sequence of the dsRNA is an important determinant not only of efficacy against a target organism but also the level of taxonomic specificity that can be achieved (Huvenne and Smagghe 2010). As demonstrated by Whyard et al. (2009), insect gene targets with a highly conserved sequence can be selectively silenced when no shared 19–21 nt sequence is present. Target sequences that diverge rapidly and significantly across taxa from the target insect will reduce the probability of an effect to non-target arthropods (Whyard et al. 2009). To illustrate the divergence of the *Snf7* sequence, a multispecies sequence alignment is presented in Fig. 2. The species selected for this alignment were chosen based upon their phylogenetic relationship to WCR. The alignment compares the *Snf7* 240 nt orthologs of selected chrysomelid beetles to DvSnf7. As a comparator, the *Snf7* orthlog from *T. castaneum* was included to further demonstrate the divergence of sequence outside the Chrysomelidae family. From these alignments, a phylogenetic tree was developed based upon the percent shared sequence identity (Fig. 3). The tree based upon the *Snf7* 240 nt orthologs follows the established phylogenetic relationships for coleopterans as reported in Clark et al. (2001), Hunt et al. (2007), and Gillespie et al. (2008) supporting the selection of these species as relevant representatives of the subfamilies examined in bioassays. The 240 nt sequence of *Snf7* diverges rapidly across insect taxa within the Chrysomelidae and the divergence of the sequence can be seen at the subfamily and tribal level. From an analysis of the percent shared sequence to DvSnf7, along with the number of 21 nt matches or longest contiguous sequence match for each *Snf7* ortholog, a clear reduction in the percent shared identity to DvSnf7 is evident as phylogenetic distance increases (Clark et al. 2001; Gillespie et al. 2008; Table 2). Additionally, the reduction in the number of 21 nt matches shown in Table 2 follows the phylogenetic tree developed for *Snf7* (Fig. 3), and is in agreement with the phylogenetic tree developed by Clark et al. (2001) for *Diabrotica* and *Acalymma* spp. using mitochondrial DNA of Cytochrome oxidase subunit 1 and the entire second internal transcribed spacer region of nuclear ribosomal DNA. Interestingly, both the *C. trifurcata* and *G. calamariensis* *Snf7* sequences possess 90.8 % shared identity with DvSnf7; however, the number of 21 nt matches is 18 and 3, respectively. This difference in the number of 21 nt matches of these two 240 nt *Snf7* orthologs reflects the location of the specific nucleotide mismatches (e.g., single nucleotide polymorphisms (SNPs)).

Heterospecific dsRNA has been used as a powerful method in diet bioassays to examine taxonomic specificity (Baum et al. 2007; Whyard et al. 2009; Burand and Hunter 2013). In this investigation, representatives of the subfamily Galerucinae were examined by indirect feeding bioassays with the WCR and CPB as model systems. Significant activity was only observed with sequences where ≥21 nt contiguous matches to the target gene sequence were present (Tables 3, 4) regardless of the number of potential 21 nt matches. These results are consistent with results from Whyard et al. (2009), where it was also demonstrated that a 19–21 nt or greater shared sequence in the target gene was required to achieve activity. We conducted diet bioassays where high concentrations of heterospecific dsRNAs from *M. ochroloma* and *C. quadrigemina* containing single 19 nt matches to DvSnf7 dsRNA were fed to WCR at 500 and 5,000 ng/mL diet, respectively, and no significant impact on survival was observed. Bolognesi et al. (2012) reported that a single 21 nt match in a neutral carrier of 240 nt induced toxicity in a highly susceptible insect (*D. undecimpunctata howardi*) at a concentration that was 200 times less than the concentration of *C. quadrigemina* -specific dsRNA fed to WCR. These results indicate that this diet bioassay methodology had sufficient sensitivity to detect potential adverse effects from a 19 nt match if it had biological activity.

**Table 3:** Summary of results from heterospecific *Snf7* dsRNAs fed to WCR in 12-day continuous exposure bioassays

Species from which dsRNA was synthesized	dsRNA concentration fed to WCR (ng/mL)	Control survival %	dsRNA treatment survival %	Statistical significance	Activity	21 nt matches
*A. vittatum*	1,000	90	8	*p* < 0.05	Active	Yes
*C. trifurcata*	500	76	15	*p* < 0.05	Active	Yes
*G. calamariensis*	5,000	89	13	*p* < 0.05	Active	Yes
*M. ochroloma*	500	76	72	*p* > 0.05	Not Active	No
*C. quadrigemina*	5,000	94	88	*p* > 0.05	Not Active	No

The concentrations of heterospecific dsRNAs were prepared at 100–1,000 times the conspecific dsRNA 12-day LC_50_ for WCR (as reported in Bolognesi et al. 2012)

**Table 4:** Summary of results from heterospecific *Snf7* dsRNAs fed to CPB in 12-day continuous exposure bioassays

Species from which dsRNA was synthesized	dsRNA concentration fed to CPB (ng/mL)	Control survival %	dsRNA treatment survival %	Statistical significance	Activity	21 nt matches
*A. vittatum*	1,000	84	88	*p* > 0.05	Not Active	No
*C. trifurcata*	1,500	92	91	*p* > 0.05	Not Active	No
*G. calamariensis*	5,000	94	38	*p* < 0.05	Active	Yes
*M. ochroloma*	5,000	92	20	*p* < 0.05	Active	Yes
*C. quadrigemina*	5,000	92	30	*p* < 0.05	Active	Yes

The concentrations of heterospecific dsRNAs were prepared at 50–250 times the conspecific dsRNA 12-day LC_50_ for CPB

The demonstrated percent shared identity and the presence of 21 nt matches necessary for toxicity in insects is important to help determine the spectrum of activity, and may also be useful in determining the potential for an insect to develop resistance to an ingested dsRNA via SNPs (Auer and Frederick 2009). Data from this current study demonstrates that a heterospecific dsRNA containing 22 SNPs (91 % shared sequence identity) resulting in only eighteen (*C*. *trifurcata*) and three (*G*. *calamariensis*) 21nt matches over the 240 nt length of DvSnf7 dsRNA is still toxic to WCR at a relatively high discriminating concentration (Tables 2, 3). Similar to the lack of substantial *Snf7* polymorphisms observed in rice, *Oryza sativa* L. (Mather et al. 2007; ncbi.nlm.nih.gov/popset/160217277), and *Drosophila melanogaster* (Chmp1; Mackay et al. 2012; ncbi.nlm.nih.gov/protein/AAF49241), preliminary analyses of field populations of WCR from multiple geographic regions shows only 1–2 SNPs in the DvSnf7 240 nt targeted sequence per population, with each population having unique sets of SNPs (L. Flagel, Monsanto, St. Louis, MO, unpublished data). Therefore, spontaneous nt mutations within the DvSnf7 open reading frame (ORF) adequate to confer resistance in WCR to plants expressing an effective dose of DvSnf7 against neonate WCR seem unlikely (Bolognesi et al. 2012; Head and Greenplate. 2012).

## Conclusions

Selecting test species based upon their phylogenetic relationship to the target organism is a powerful approach to characterize the spectrum of activity for genetically engineered crops, as this focuses testing to those species most likely to be susceptible due to sequence similarity (Romeis et al. 2013). The results from direct feeding bioassays demonstrate that biological activity of DvSnf7 dsRNA was only evident in a subset of beetles within the family Chrysomelidae and more specifically, the subfamily Galerucinae. Results from indirect feeding assays with the WCR and CPB demonstrate that a ≥21 nt contiguous sequence is required to observe biological activity in a sensitive insect and that a DvSnf7 ortholog sequence from a related species with as little as three 21 nt sequence matches can induce significant activity at a discriminating high dose. Further, there are additional barriers to achieving an RNAi-mediated effect in insects and consequently not all species are susceptible to ingested dsRNA or may not be susceptible at environmentally relevant exposure concentrations (Huvenne and Smagghe 2010; Terenius et al. 2011; Allen and Walker 2012). Because of these barriers, bioinformatics analyses can provide supplemental information to the results from insect bioassays but cannot be reliably used as a standalone to predict the presence of RNAi activity. When bioinformatics data for non-target arthropods is available, and indicates that the minimum sequence requirements for RNAi activity are not met, then toxicity testing is not necessary as the likelihood of adverse effects is low. Due to the high specificity of the DvSnf7 dsRNA characterized in these bioassays, only a limited subset of the Chrysomelidae family are likely susceptible to DvSnf7 dsRNA. Therefore, the likelihood of adverse effects to non-target arthropods from a realistic environmental exposure to DvSnf7 dsRNA is predicted to be extremely low. These results along with other published data (Bolognesi et al. 2012) also suggest that resistance to DvSnf7 dsRNA due to SNPs in the target sequence of 240 nt is highly unlikely.

## Electronic supplementary material

Below is the link to the electronic supplementary material.

Supplementary material 1 (PDF 60 kb)

Supplementary material 2 (PDF 34 kb)
