# Supplementary material for: Characterization of the spectrum of insecticidal activity of a double-stranded RNA with targeted activity against Western Corn Rootworm (Diabrotica virgifera virgifera LeConte)
Source: Transgenic Res. 2013 Jun 8;22(6):1207–22. doi: 10.1007/s11248-013-9716-5 (PMC3835954; doi:10.1007/s11248-013-9716-5)
Supplement: Supplementary file 1 — Supplementary material 1 (PDF 60 kb) [file 11248_2013_9716_MOESM1_ESM.pdf]

Transgenic Research

Characterization of the spectrum of insecticidal activity of a double-stranded RNA with targeted activity against Western Corn Rootworm (*Diabrotica virgifera virgifera* LeConte)

Pamela M. Bachman*[1], Renata Bolognesi[2], William J. Moar[1], Geoffrey M. Mueller[1], Mark S. Paradise[1], Parthasarathy Ramaseshadri[2] , Jianguo Tan[1], Joshua P. Uffman[1], JoAnne Warren[1], B. Elizabeth Wiggins[2], and Steven L. Levine[1]

1. Monsanto Company, 800 N Lindbergh Blvd., St. Louis, MO 63167, USA
2. Monsanto Company, 700 Chesterfield Parkway W, Chesterfield, MO 63017, USA

*****Corresponding author**:** Pamela M. Bachman, pamela.m.bachman@monsanto.com

Online Resource 1 Fig A-F (A) *O. insidiosus* percent development to adult for test (5,000 ng DvSnf7/g diet) and control treatments (n=40) were comparable with mean emergence times of 10.7 ± 0.1 and 10.6 ± 0.1 days, respectively. (B) *N. vitripennis* percent survival after 20 days of exposure was not significantly different between the test (5,000 ng DvSnf7/mL diet) and control treatments ($p > 0.05$). The concurrent positive control of 100 µg potassium arsenate/mL diet treatment resulted in a significant effect on survival ($p < 0.05$). Each treatment consisted of three replicates of 25 wasps each. (C) *S. frugiperda* mean body weight in the test (500 ng DvSnf7/mL diet) and control treatments (n=32) were not significantly different after 8 days of exposure ($p > 0.05$). (D) *H. zea* mean body weight in the test (5,000 ng DvSnf7/mL diet) and control treatments were not significantly different after 10 and 12 days of exposure ($p > 0.05$). Each treatment consisted of three replicates of 16 individually housed larvae.(E) *O. nubilalis* percent survival after 12 days of exposure was not significantly different in the test (5,000 ng DvSnf7/mL diet) and control treatments ($p > 0.05$). Each treatment consisted of three replicates of 16 individually housed larvae. (F) *O. nubilalis* mean body weight in the test (5,000 ng DvSnf7/mL diet) and control treatments were not significantly different after 12 days of exposure ($p > 0.05$)

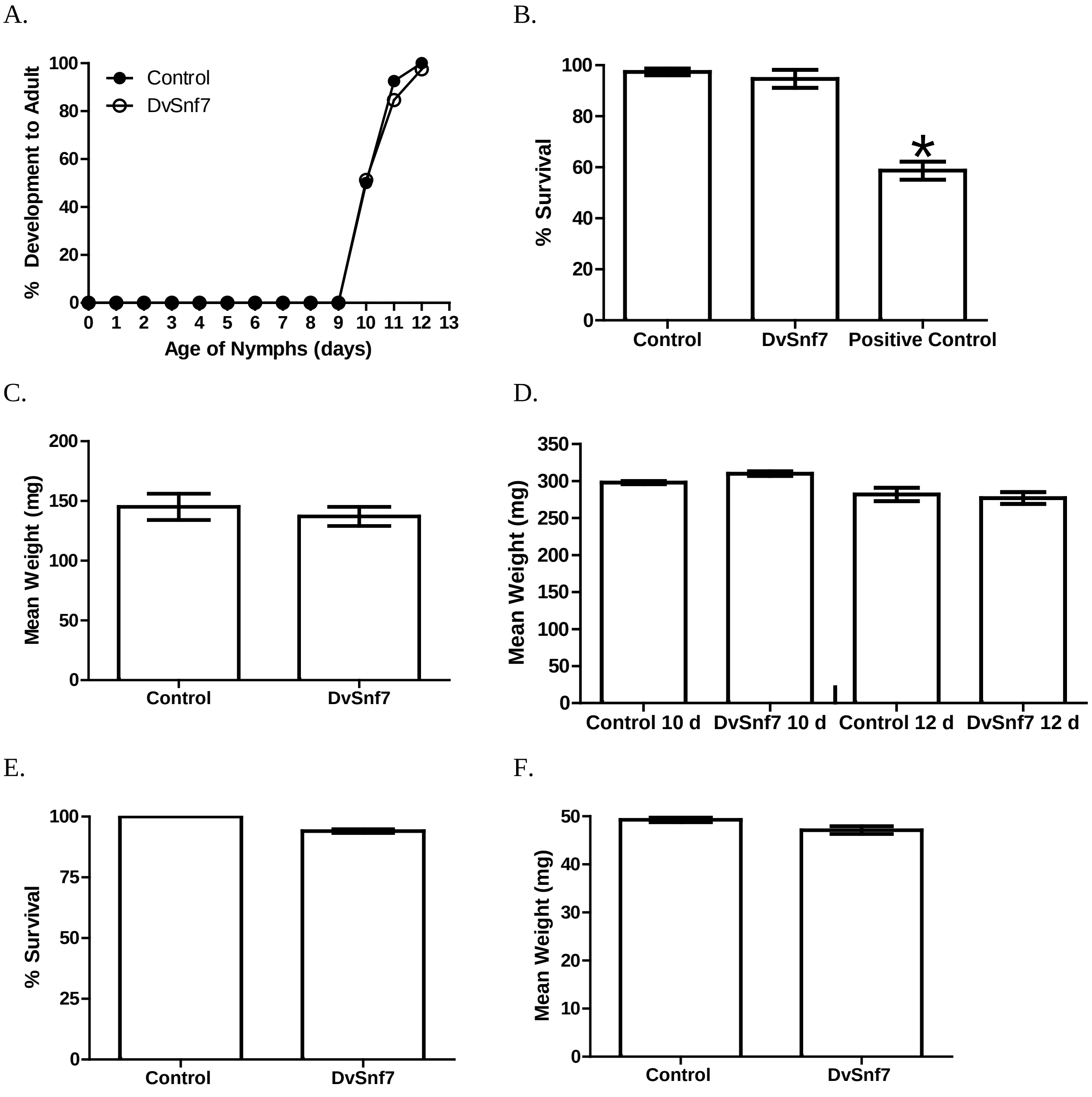
A.
% Development to Adult
Control
DvSnf7
Age of Nymphs (days)
0 1 2 3 4 5 6 7 8 9 10 11 12 13
0 20 40 60 80 100
B.
% Survival
0 20 40 60 80 100
*
Control
DvSnf7
Positive Control
C.
Mean Weight (mg)
0 50 100 150 200
Control
DvSnf7
D.
Mean Weight (mg)
0 50 100 150 200 250 300 350
Control 10 d
DvSnf7 10 d
Control 12 d
DvSnf7 12 d
E.
% Survival
0 25 50 75 100
Control
DvSnf7
F.
Mean Weight (mg)
0 10 20 30 40 50
Control
DvSnf7

Online Resource 1 Fig G-K (G) *B. mori* percent survival for after 14 days of exposure was not significantly different in the test (5,000 ng DvSnf7/mL leaf dip solution) and control treatments ($p > 0.05$). The concurrent positive control treatment with Cry1Ab resulted in a significant effect on survival. Each treatment consisted of five replicates of 10 larvae each. (H) *B. mori* mean body weight in the test (5,000 ng DvSnf7/mL leaf dip solution) and control treatments were not significantly different after 14 days of exposure ($p > 0.05$). The concurrent positive control treatment with Cry1Ab resulted in a significant effect ($p < 0.05$) on mean body weight of surviving larvae noted with an asterisk. (I) Concentration response curves for WCR and *D. undecimpunctata howardi* (SCR) in 12-day diet bioassays; mean LC_{50} values of 4.4 ng DvSnf7 dsRNA/mL and 1.2 ng DvSnf7 dsRNA/mL diet as reported by Bolognesi et al. (2012). (J) *C. maculata* percent development to adult for the test (3,000 ng DvSnf7/g diet) and control treatments (four replicates of 20 larvae each) were the same with mean emergence times of 19.6 ± 1.2 and 19.6 ± 1.5 days, respectively. (K) *C. maculata* mean adult body weights in the test (3,000 ng DvSnf7/g diet) and control treatments were not significantly different after 24 days of exposure ($p > 0.05$)

G.

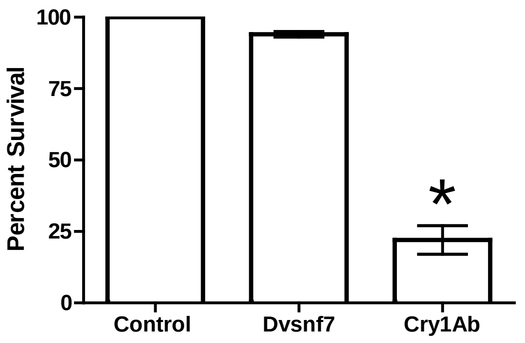
Percent Survival
100
75
50
25
0
Control
Dvsnf7
Cry1Ab
*

H.

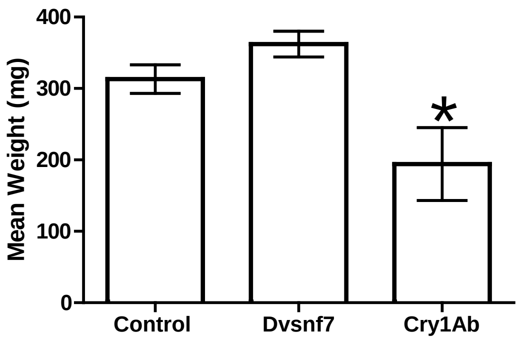
Mean Weight (mg)
400
300
200
100
0
Control
Dvsnf7
Cry1Ab
*

I.

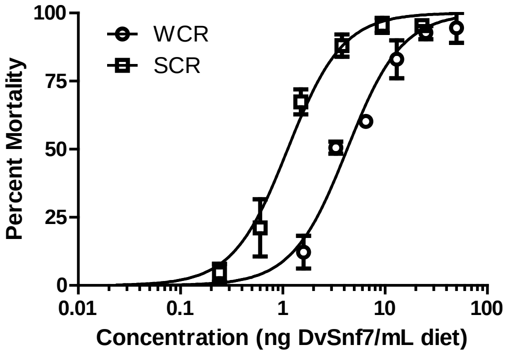
Percent Mortality
100
75
50
25
0
WCR
SCR
0.01
0.1
1
10
100
Concentration (ng DvSnf7/mL diet)

J.

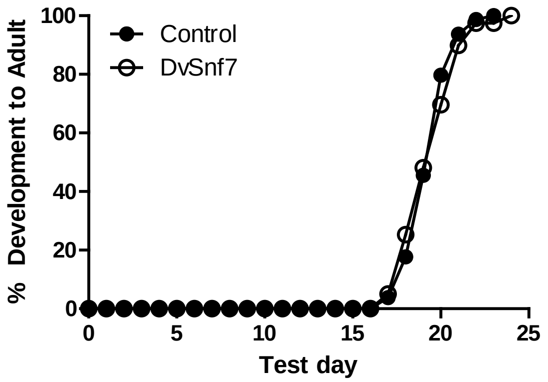
% Development to Adult
100
80
60
40
20
0
Control
DvSnf7
0
5
10
15
20
25
Test day

K.

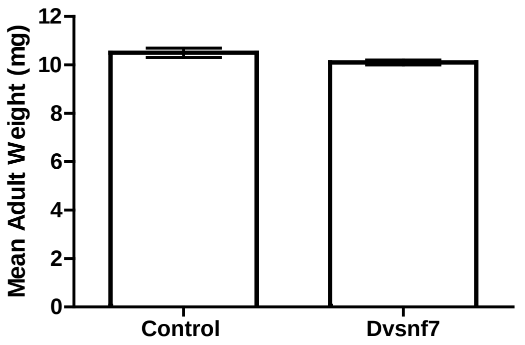
Mean Adult Weight (mg)
12
10
8
6
4
2
0
Control
Dvsnf7

Online Resource 1 Fig L-Q (L) Percent *E. varivestis* survival after 28 days of exposure was the same in the test (3,000 ng DvSnf7/mL diet) and control treatments. The concurrent positive control treatments of 14 and 28 µg potassium arsenate/g diet resulted in a significant and concentration dependent effect on *E. varivestis* survival ($p < 0.05$). Each treatment consisted of three replicates of 16 larvae each. (M) Mean *E. varivestis* body weight in the test (3,000 ng DvSnf7/mL diet) and control treatments were not significantly different after 28 days of exposure ($p > 0.05$). (N) Percent *P. chalcites* survival in the test (5,000 ng DvSnf7/g diet) and control treatments over the 35 day exposure study were 89% and 90%, respectively. The concurrent positive control of 200 µg potassium arsenate/g diet resulted in a significant effect on *P. chalcites* survival ($p < 0.05$). Each treatment consisted of three replicates of 25 to 30 larvae each. (O) *P. chalcites* percent development to adult for the test (5,000 ng DvSnf7/g diet) and control treatments were comparable with mean emergence times of 29 ± 1 and 30 ± 1 day, respectively. (P) Mean *P. chalcites* adult body weight in the test (5,000 ng DvSnf7/g diet) and control treatments were not significantly different ($p > 0.05$) in a 35 day exposure study

L

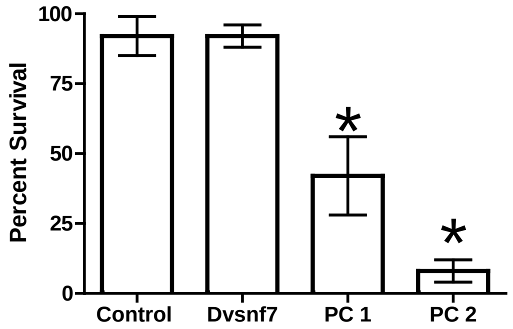
Percent Survival
100
75
50
25
0
Control
Dvsnf7
PC 1
PC 2
*
*

M.

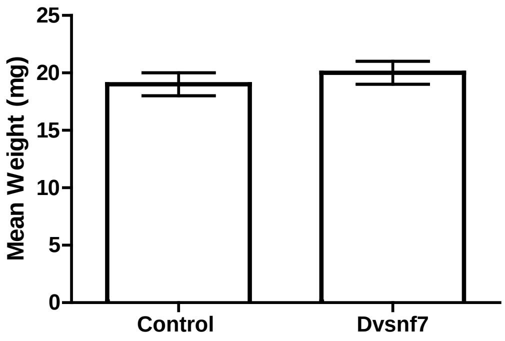
Mean Weight (mg)
25
20
15
10
5
0
Control
Dvsnf7

N.

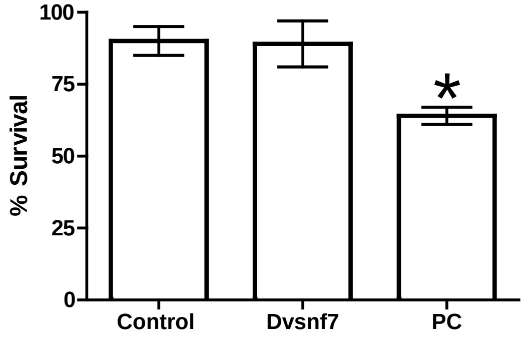
% Survival
100
75
50
25
0
Control
Dvsnf7
PC
*

O.

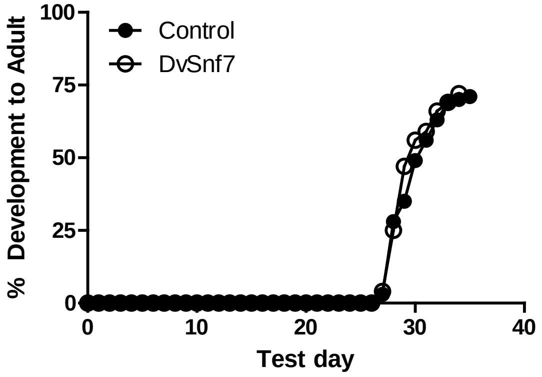
% Development to Adult
100
75
50
25
0
Control
DvSnf7
0
10
20
30
40
Test day

P.

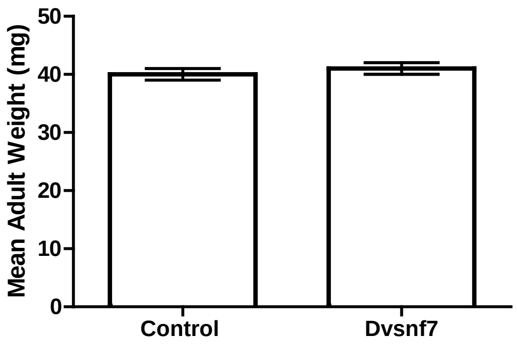
Mean Adult Weight (mg)
50
40
30
20
10
0
Control
Dvsnf7
