# Supplementary material for: Characterization of the spectrum of insecticidal activity of a double-stranded RNA with targeted activity against Western Corn Rootworm (Diabrotica virgifera virgifera LeConte)
Source: Transgenic Res. 2013 Jun 8;22(6):1207–22. doi: 10.1007/s11248-013-9716-5 (PMC3835954; doi:10.1007/s11248-013-9716-5)
Supplement: Supplementary file 2 — Supplementary material 2 (PDF 34 kb) [file 11248_2013_9716_MOESM2_ESM.pdf]

Transgenic Research

Characterization of the spectrum of insecticidal activity of a double-stranded RNA with targeted activity against Western Corn Rootworm (*Diabrotica virgifera virgifera* LeConte)

Pamela M. Bachman*[1], Renata Bolognesi[2], William J. Moar[1], Geoffrey M. Mueller[1], Mark S. Paradise[1], Parthasarathy Ramaseshadri[2] , Jianguo Tan[1], Joshua P. Uffman[1], JoAnne Warren[1], B. Elizabeth Wiggins[2], and Steven L. Levine[1]

1. Monsanto Company, 800 N Lindbergh Blvd., St. Louis, MO 63167, USA
2. Monsanto Company, 700 Chesterfield Parkway W, Chesterfield, MO 63017, USA

***Corresponding author:** Pamela M. Bachman, pamela.m.bachman@monsanto.com

Online Resource 2 FASTA alignment of the *N. vitripennis* 240 nt *Snf7* ortholog (NvSnf7) to the DvSnf7 sequence shows no contiguous sequence match greater than 14 nt and an overall shared sequence identity of 71%. The alignment was created using FASTA version 36.3.5c (Pearson and Lipman, 1988). The NvSnf7 sequence is UniGene sequence Nvi#S49011431

```
     240       230       220       210       200       190
DvSnf7 GCAAAGAAAAATGCGTCGAAAAATAAAAGAGTTGCACTCCAAGCCCTCAAAAAGAAGAAA
       :: : ::: :: :   : :: :: : ::::: :::  : :::::  : :: : :::::: 
NvSnf7 GCCAGGAAGAACGGCACCAAGAACAGAAGAGCTGCCATTCAAGCATTAAAGAGGAAGAAG
              160       170       180       190       200       210

     180       170       160       150       140       130
DvSnf7 CGATTGGAAAAGACCCAACTACAAATAGATGGAACCCTTACAACTATTGAAATGCAGAGG
       ::::  :: :::   :  :  ::::: :::::::: ::  : :: ::::: :::::::: 
NvSnf7 CGATATGAGAAGCAACTTCAGCAAATCGATGGAACTCTATCCACGATTGAGATGCAGAGA
              220       230       240       250       260       270

     120       110       100        90        80        70
DvSnf7 GAAGCCCTCGAAGGAGCTAGCACAAATACTGCTGTATTAGATTCTATGAAAAATGCTGCA
       ::::: :: ::: : :: :  ::::::::::::::  :     : :::::::::::::: 
NvSnf7 GAAGCACTGGAAAGTGCAAATACAAATACTGCTGTTCTTACCACGATGAAAAATGCTGCT
              280       290       300       310       320       330

      60        50        40        30        20        10
DvSnf7 GATGCCCTTAAGAAAGCTCATAAGAATTTGAATGTAGATGATGTTCACGATATCATGGAT
       :::::::::::   ::::::: :  :  :: :::: ::  : ::::::::::: ::::::
NvSnf7 GATGCCCTTAAAGCAGCTCATCAACACATGGATGTTGACCAAGTTCACGATATGATGGAT
              340       350       360       370       380       390
```

Online Resource 3 FASTA alignment of the *B. mori* 240 nt *Snf7* ortholog (BmSnf7) to DvSnf7 dsRNA shows no contiguous sequence match greater than 15 nt and an overall shared sequence identity of 66%. The alignment was created using FASTA version 36.3.5c (Pearson and Lipman, 1988). The BmSnf7 sequence is UniGene sequence Bmo#S58881086

```
     240       230       220       210       200       190
DvSnf7 GCAAAGAAAAATGCGTCGAAAAATAAAAGAGTTGCACTCCAAGCCCTCAAAAAGAAGAAA
       :: : :::: :::   : :: :: ::::::: :::  :   ::: :::::   ::::::
BmSnf7 GCCAGGAAACATGGCACTAAGAACAAAAGAGCTGCCATTGCAGCTCTCAAGCGGAAGAAG
             290       300       310       320       330       340

     180       170        160       150       140       130
DvSnf7 CGATTGGAAAAG-ACCCAACTACAAATAGATGGAACCCTTACAACTATTGAAATGCAGAG
        :::  :: ::: : :  ::: ::::::::::: ::::: ::    :::::    :: ::
BmSnf7 AGATACGAGAAGCAACTTACT-CAAATAGATGGGACCCTCACTCAGATTGAGGCCCAAAG
             350       360        370       380       390       400

      120       110       100        90        80        70
DvSnf7 GGAAGCCCTCGAAGGAGCTAGCACAAATACTGCTGTATTAGATTCTATGAAAAATGCTGC
       :::::: :: ::::: :: :  :: ::: :    :::::  :  :::::    : :: ::
BmSnf7 GGAAGCGCTAGAAGGTGCCAATACCAATGCCCAAGTATTGAACACTATGCGCGAGGCAGC
              410       420       430       440       450       460

       60        50        40        30        20        10
DvSnf7 AGATGCCCTTAAGAAAGCTCATAAGAATTTGAATGTAGATGATGTTCACGATATCATGGAT
         ::::: : :::   :: :: ::  :  :  :::: ::  : :: :::::::::::::::
BmSnf7 TAATGCCATGAAGCTCGCACACAAAGACATTGATGTGGACAAAGTACACGATATCATGGAT
              470       480       490       500       510       520
```

Online Resource 4 FASTA alignment of the *T. castaneum* 240 nt *Snf7* ortholog (TcSnf7) to the DvSnf7 dsRNA sequence showed no contiguous sequence match greater than 11 nt and an overall 72% shared sequence identity. The alignment was created using FASTA version 36.3.5c (Pearson and Lipman, 1988). The TcSnf7 sequence is UniGene sequence Tca#S32316245

```
     240       230       220       210       200       190
DvSnf7 GCAAAGAAAAATGCGTCGAAAAATAAAAGAGTTGCACTCCAAGCCCTCAAAAAGAAGAAA
       :: :::::::: ::::::::::: ::: :::  ::  :::: :::::::: : :::::::
TcSnf7 GCGAAGAAAAACGCGTCGAAAAACAAACGAGCGGCCATCCAGGCCCTCAAGAGGAAGAAA
        290       300       310       320       330       340

     180       170       160       150       140       130
DvSnf7 CGATTGGAAAAGACCCAACTACAAATAGATGGAACCCTTACAACTATTGAAATGCAGAGG
       :: :  ::::::   :  :  :: :: ::::: ::::: :  :: :: :: :::::: ::
TcSnf7 CGCTACGAAAAGCAGCTCCAGCAGATCGATGGCACCCTCAGCACCATCGAGATGCAGCGG
        350       360       370       380       390       400

     120       110       100        90        80        70
DvSnf7 GAAGCCCTCGAAGGAGCTAGCACAAATACTGCTGTATTAGATTCTATGAAAAATGCTGCA
       :: :::::::: :: :: : ::: :: :: :: ::: :  :  : :::::::: :: ::
TcSnf7 GAGGCCCTCGAGGGGGCCAACACCAACACAGCCGTACTCAAAACGATGAAAAACGCAGCG
        410       420       430       440       450       460

      60        50        40        30        20        10
DvSnf7 GATGCCCTTAAGAAAGCTCATAAGAATTTGAATGTAGATGATGTTCACGATATCATGGA
       :: ::::: :: :: :: ::    ::  :: :::: ::::: :: ::: :::: :::::
TcSnf7 GACGCCCTCAAAAATGCCCACCTCAACATGGATGTTGATGAGGTCCACAATATGATGGA
        470       480       490       500       510       520
```

Reference

Pearson WR, Lipman DJ (1988) Improved tools for biological sequence comparison. Proc Natl Acad Sci 85:2444-2448.
